# Successful rituximab treatment for severe rapidly progressive interstitial lung disease with anti-MDA5 antibody-positive juvenile dermatomyositis: a case report and literature review

**DOI:** 10.1186/s12969-022-00723-5

**Published:** 2022-08-04

**Authors:** Kentaro Nishi, Masao Ogura, Naotaka Tamai, Naofumi Gima, Kentaro Ide, Goro Koinuma, Koichi Kamei, Shuichi Ito

**Affiliations:** 1 Division of Nephrology and Rheumatology, National Center for Child Health and Development, Tokyo, Japan; 2 Division of Pulmonology, National Center for Child Health and Development, Tokyo, Japan; 3 Division of Critical Care Medicine, National Center for Child Health and Development, Tokyo, Japan; 4 Department of Pediatrics, Yokohama City University Graduate School of Medicine, 3-9, Fukuura, Kanazawa-ku, Kanagawa 236-0004 Yokohama, Japan

**Keywords:** Dermatomyositis, Anti-melanoma differentiation-association gene 5 antibody, Extracorporeal membrane oxygenation, Plasma exchange, Prone position, Children

## Abstract

**Background:**

Rapidly progressive (RP) interstitial lung disease (ILD) is a life-threatening complication of juvenile dermatomyositis (JDM); however, it is generally refractory to treatment; to the best of our knowledge, no evidence-based treatment has been established for RP-ILD yet. We present the case of a 2-year-old girl with RP-ILD who showed resistance to treatment with methylprednisolone, cyclosporine A, cyclophosphamide, immunoglobulin, and plasma exchange (PE) and was finally treated with extracorporeal membrane oxygenation. We further present a literature review of 18 cases of JDM with RP-ILD.

**Case presentation:**

A 2-year-old girl presented with malar rash, mild muscle weakness, and weight loss for a few months before admission. She had a history of dry cough and dyspnea for a few days, followed by rapid respiratory failure. The patient was diagnosed with JDM with RP-ILD through physical examination (malar rashes and Gottron’s sign) and based on the finding of myositis on femoral magnetic resonance imaging, elevated levels of serum muscle enzymes, positive anti-melanoma differentiation-association gene 5 (MDA5) antibody (> 7,500 index), elevated level of Krebs von den Lungen-6 glycoprotein (KL-6; 3,420 U/mL), and extensive ground-glass opacities with consolidation in the bilateral lungs on chest high-resolution computed tomography. She received combination therapy, including methylprednisolone pulse therapy, followed by oral prednisolone and intravenous cyclosporine A, cyclophosphamide, and immunoglobulin. On day 11 of hospitalization, she was placed on ventilation support and PE was initiated. However, her respiratory condition continued to deteriorate and veno-venous extracorporeal membrane oxygenation was started on day 24 of hospitalization. Rituximab was administered on day 28. After 2 weeks of rituximab therapy initiation, her respiratory condition showed gradual improvements. Eventually, on day 52 of hospitalization, the patient could be weaned off extracorporeal membrane oxygenation. Finally, she was discharged with minimal ventilation support and no neurological complications 11 months after admission.

**Conclusions:**

Our literature review suggest that JDM with RP-ILD has a high mortality rate. In JDM, rituximab may be a promising treatment option for RP-ILD. In the future, the efficacy of rituximab in the early phases of ILD should be investigated.

## Background

Juvenile dermatomyositis (JDM) is a rare autoimmune disease in children [1]; this is complicated by interstitial lung disease (ILD) in 8%–53% of cases in Japan [2, 3]. In particular, rapidly progressive ILD (RP-ILD) is a rare but life-threatening complication. JDM-associated RP-ILD is generally refractory to treatment, and to the best of our knowledge, no evidence-based treatment has been established yet. Despite potent combination therapy including methylprednisolone pulse therapy (MPT), calcineurin inhibitor, and/or intravenous cyclophosphamide (IVCY), as well as intravenous immunoglobulin (IVIG), the prognosis of JDM-associated RP-ILD remains unfavorable [1]. Although, the efficacy of plasma exchange (PE) or rituximab (RTX) therapy has been reported in a small number of in adult patients with dermatomyositis with associated RP-ILD, the reports in children are limited [4–6].

Anti-melanoma differentiation-association gene 5 (MDA5) antibody has a significant correlation with JDM-associated ILD [4]. In particular, patients with JDM with a high level of anti-MDA 5 antibody are likely to develop RP-ILD [3, 7]. Here we report the case of a 2-year-old girl with RP-ILD with anti-MDA5 antibody-positive JDM who exhibited resistance to MPT, cyclosporine A (CyA), IVCY, IVIG, and PE. She finally required veno-venous extracorporeal membrane oxygenation (VV-ECMO); ultimately, RTX therapy helped her achieve remission and survive.

## Case presentation

A previously healthy 2-year-old girl presented with malar rash, mild muscle weakness, and weight loss for a few months before admission. Her medical and family histories were unremarkable. She was previously admitted to another hospital owing to complaints of dry cough and dyspnea, which developed a few days ago. She was diagnosed with JDM based on the European League Against Rheumatism/American College of Rheumatology (EULAR/ACR) classification criteria for adult and juvenile idiopathic inflammatory myopathies (progressive muscle weakness of the proximal lower extremities, Gottoron papules, Gottoron’s sign, and elevated serum levels of muscle enzymes). Femoral magnetic resonance imaging suggested myositis. Her percutaneous oxygen saturation was 96% on oxygen via nasal canula at 2 L/min. After 6 days since diagnosis, the patient’s respiratory state rapidly deteriorated and she was transferred to our intensive care unit with a diagnosis of JDM-associated RP-ILD.

**Fig. 1:**
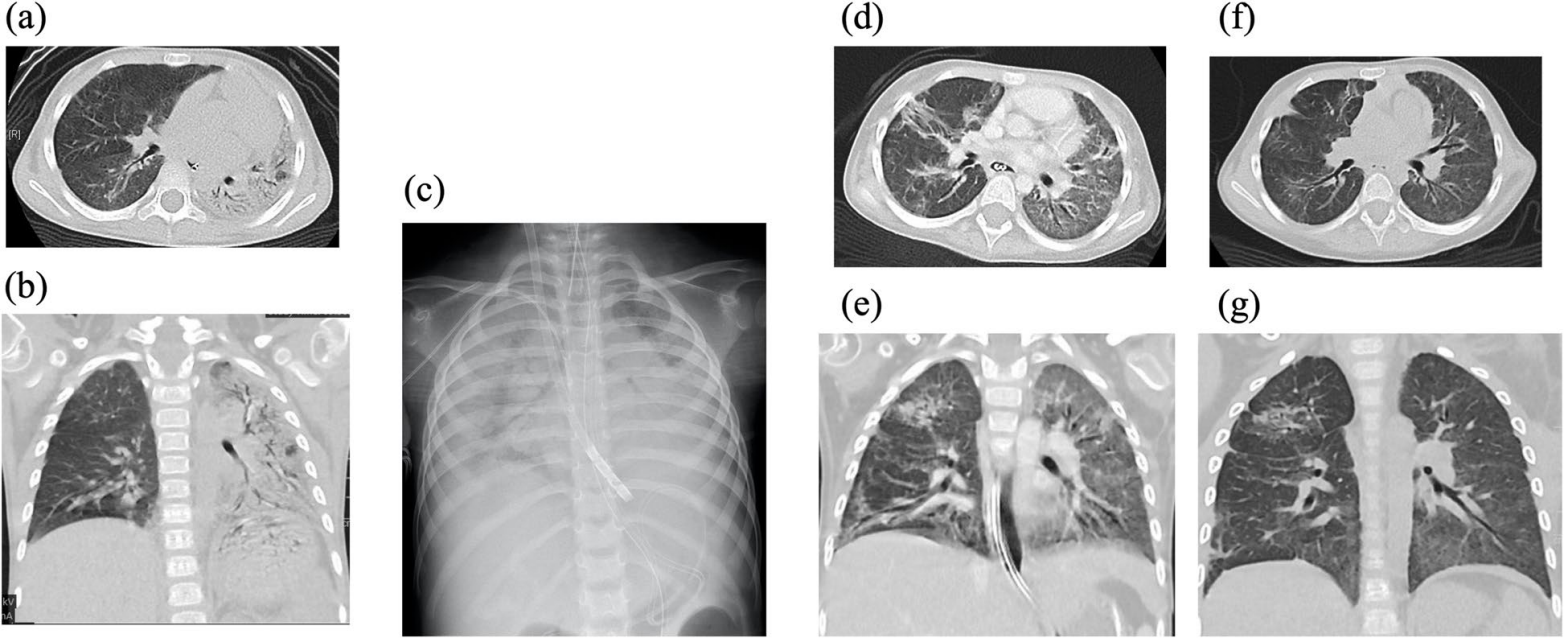
Chest computed tomography of the patient. **a** and **b** Lung computed tomography (CT) scan performed at admission showing extensive ground-glass opacities with consolidation in bilateral lungs, significant on the left side. **c** Chest X-ray performed upon the initiation of veno-venous extracorporeal membrane oxygenation showing massive bilateral areas of consolidation. **d** and **e** Lung CT scan performed after treatment on day 78 of hospitalization showing improvement in aeration and resolution of consolidation. Ground-glass opacities were enlarged, and traction bronchiectasis was evident. **f** and **g** Lung CT scan performed at 11 months after admission showing improvement in both lungs

Upon admission to our institution, she presented with low-grade fever (37.4 °C), tachycardia (158 beats per min), tachypnea (56 breaths per min), with an oxygen saturation of 86%–96% on oxygen mask (8 L/min). Her height and body weight were 92 cm and 11.2 kg, respectively; she lost 1.8 kg in 5 months. Malar rashes and Gottron’s sign (i.e., papules on knuckles and elbows) were noted. The laboratory results showed the following levels of the parameters studied: creatinine kinase, 22 (normal: 43–270) U/L; alanine transaminase, 69 (normal: 24–44) U/L; aspartate transaminase, 49 (normal: 9–30) U/I; lactate dehydrogenase (LDH): 576 (normal: 190–365) U/L; aldolase: 13.5 (normal: < 6.1) U/L; Krebs von den Lungen-6 glycoprotein (KL-6), 3,420 (normal: < 500) U/mL; anti-MDA5 antibody, > 7,500 (normal: < 500) index; interleukin-6, 5.2 (normal: < 6) pg/mL, and Interleukin-18, 1102.6 (normal: < 500) pg/mL. Anti–Ro52 antibody could not be measured. Other autoantibodies and infection-related tests were all negative. PaO_2_ was 68.7 mmHg on oxygen mask (8 L/min). Chest high-resolution computed tomography (CT) revealed extensive ground-glass opacities with consolidation in the bilateral lungs, higher in the left lung; these findings are consistent with a diffuse alveolar damage pattern (Fig. 1a and b). Her respiratory condition was too severe to use tools such as the Childhood Myositis Assesment Scale or Manual Muscle Testing-8. Therefore, we assessed disease activity and response to treatment for myositis using the results of various blood test parameters such as the levels of muscle enzymes, liver enzymes, and LDH. Disease activity and response to treatment for ILD were assesed using KL-6 level, anti-MDA5 antibody level, her respiration condition, and chest X-ray findings. The level of creatinine kinase did not increase during the disease course, which is commonly observed in anti-MDA-5 antibody-related JDM. LDH and KL-6 levels represented important markers before PE initiation.

**Fig. 2:**
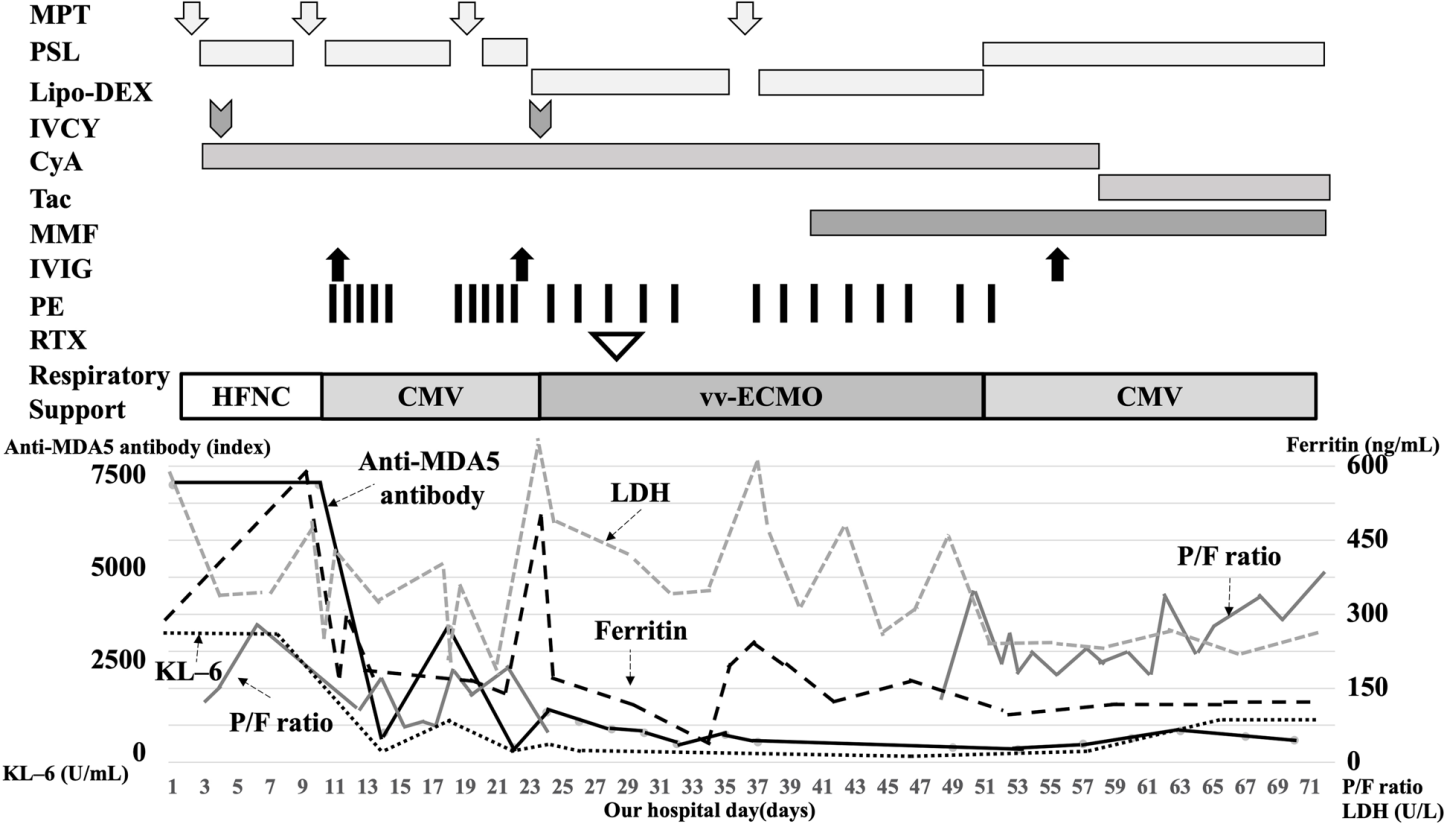
Clinical course and treatment. Clinical course and treatment of the patient. MPT, methylprednisolone pulse therapy; PSL, prednisolone; Lipo-Dex, liposteroid dexamethasone; IVCY, intravenous cyclophosphamide; CyA, cyclosporine A; Tac, tacrolimus; MMF, mycophenolate mofetil; IVIG, intravenous immunoglobulin; PE, plasma exchange; RTX, rituximab; HFNC, High-flow nasal cannula; CMV, conventional mechanical ventilation; VV-ECMO, veno-venous extracorporeal membrane oxygenation; KL–6, Krebs von den Lungen-6; MDA5, melanoma differentiation-association gene 5; P/F ratio, PaO2/FiO2 ratio

Owing to the rapid progression of her respiratory failure, the patient received combination therapy including four courses of MPT (30 mg/kg/day for 3 days per course) followed by oral prednisolone (PSL; 1 mg/kg/day) and intravenous CyA (starting with 3 mg/kg/day and adjusted to maintain trough levels at 150–200 ng/mL), two courses of IVCY (500 mg/m^2^/dose at a 1-month interval), and IVIG (2 g/kg/dose, Fig. 2). She also received trimethoprim–sulfadiazine as prophylaxis for opportunistic infections. Her respiratory condition deteriorated further, and she was placed on a ventilator support on day 11 of hospitalization; the following settings were used: mean airway pressure (MAP), 12 cmH_2_O; peak inspiratory pressure (PIP), 20 cmH_2_O;positive end-expiratory pressure (PEEP), 6 cmH_2_O; and FiO_2_, 0.8 (oxygen index: 8.0, PaO_2_/FiO_2_: 150).

Her respiratory condition worsened further, and the level of anti-MDA-5 antibody did not decrease. Thus, PE was initiated on day 11 of hospitalization (Fig. 2); a total of 23 sessions of PE were performed. Afterward, the serum level of anti-MDA5 antibody decreased temporarily but soon increased markedly. Furthermore, her respiratory status, including oxygen demand, showed no improvements.

On day 24 of hospitalization, the patient could no longer maintain an SpO_2_ of 90% at the following ventilator settings: MAP, 18 cmH_2_O; PIP, 31 cmH_2_O; PEEP, 10 cmH_2_O; FiO_2_, 1.0 (oxygen index: 20, PaO_2_/FiO_2_: 50), and nitric oxide, 5.0 ppm. We then decided to introduce VV-ECMO for life support. As her respiratory condition was unstable, we could not performe a follow-up chest CT after her admission. Plain chest radiography performed at that time showed diffuse ground-glass opacities (Fig. 1c).

After receiving approval from the institutional ethical committee (approval no. 2020–03), 375 mg/m^2^ of RTX was administered on day 28 of hospitalization. Mycophenolate mofetil (MMF) was added on day 40 of hospitalization. Respiratory physical therapy, including placement of the patient in the prone position, was continued under VV-ECMO. After 2 weeks of RTX therapy initiation, the patient’srespiratory status started to improve gradually. She was eventually weaned off ECMO on day 52 of hospitalization. Chest CT performed on day 78 of hospitalization showed improved aeration and reduced consolidation. However, the ground-glass opacities enlarged and traction bronchiectasis was also noted (Fig. 1d-e). The levele of anti-MDA-5 antibody decreased steadily after the first course of RTX, and thus additional RTX was administered at 2 and 6 months because of the recovery of B cells from peripheral blood.

No severe respiratory complications such as pneumonia, pneumothorax, pulmonary hemorrhage, or thrombosis occurred in the acute phase. We avoided high ventilation pressures above PIP 30 cmH_2_O as much as possible to avoid pneumothorax.

After 3 months of admission, the patient’s general condition improved without neurological complications. Her activities of daily living improved after continuous rehabilitation. She regained the abilities to speak, eat, walk, and bathe at 3, 4, 6, and 10 months after admission, respectively. Finally, she was discharged with minimum ventilator support 11 months after admission. At discharge, she was weaned from the ventilator for short periods; the ventilator settings were as follows: MAP, 8 cmH_2_O; PIP, 14 cmH_2_O; PEEP, 7 cmH_2_O; and FiO_2_, 0.24. Chest CT revealed remarkable improvement in both lungs at this time (Fig. 1f-g). Currently, at 5 months after discharge, the patient can withdraw from the ventilator for up to 9 h per day but requires oxygen at 0.5 L/min. Her anti-MDA5 antibody level was 450 index, and her KL-6 level was 1,150 U/mL. Her disease is now managed with PSL 4 mg/day, tacrolimus adjusted with trough levels of nearly 5.0 ng/mL, and MMF 500 mg/day.

## Discussion and conclusions

We reported the effectiveness of combined therapy including RTX for a 2-year-old girl with life-threatening RP-ILD associated with anti-MDA5 antibody-positive JDM. Although the patient’s condition deteriorated and she eventually required VV-ECMO, she was rescued with the aforementioned therapies. This case suggests that RTX is an effective option in JDM for severe and refractory RP-ILD that does not respond to current combination therapy.

Anti-MDA-5 antibody-positive JDM and adult dermatomyositis are known to be more common in Asians than in other races [8]. Notably, in Japan, 28% of children with JDM and juvenile polymyositis and 10%–48% of adults with dermatomyositis and polymyositis are positive for anti-MDA-5 antibodies [8]. Furthermore, patients with anti-MDA-5 antibody-positive JDM and adult dermatomyositis are prone to developing RP-ILD [3, 7]; in JDM specifically, RP-ILD has a poor prognosis with no established treatment. To study this further, we conducted a literature review of JDM with RP-ILD and found 18 cases (Table 1) [3, 6, 8–19]. The search was performed in July 2021 using the PubMed/Medline database. The search period was from January 2000 to July 2021. Our main search comprised terms related to “juvenile dermatomyositis” combined with the term “interstitial lung disease.” We used the following search formula:

**Table 1:** Literature review of 18 cases of RP-ILD with JDM

Reference	Age/sex/race	Clinical symptom of JDM				Interval between onset of JDM and diagnosis of ILD	Interval between diagnosis of JDM and diagnosis of ILD	Clinical presentation of RP-ILD	Blood test	
		Fever	Rash	Arthralgia	Muscle weakness				AST/ALT/LDH/CK/ALD/KL-6	Antibody (level or index)
Tosun et al. [9]	10/M/Turkish	+	+	+	+	5 weeks	2 weeks	dyspnea	240/318/3545/437/NA/NA	ND
Zulian et al. [10]	3/F/Italy	+	+	+	+	8 months	7 months	cough, dyspnea	334/NA/3,640/403/NA/NA	ND
Nonaka et al. [3, 11]	1/F/Japanese	+	+	+	+	5 months	2 months	−	178/69/1658/116/18.4/2850	Anti-Jo-1 (12.8)
Nagai et al. [12]	5/F/Japanese	+	+	−	+	1 month	2 day	cough, dyspnea	256/169/649/40/32.9/3,173	ND
Sakurai et al. [13]	9/M/Japanese	+	+	−	+	6 months	2 months	ND	207/101/564/315/16.7/5138	Anti-MDA5 (NA)
Kobayashi et al. [3, 8, 13, 15, 16]	14/F/Japanese	+	+	ND	+	ND	1 month	mild dyspnea	244/ND/ND/1435/20.5/1695	Anti-MDA5^a^
	7/M/Japanese	+	+	−	−	ND	2 months	cough, dyspnea	71/38/647/1250/9.6/2460	Anti-MDA5^a^
	7/M/Japanese	−	+	ND	+	ND	1 month	−	228/ND/ND/348/20.0/2376	Anti-MDA5^a^
Ishikawa et al. [3, 17]	10/M/Japanese	+	−	+	+	2 months	1 month	cough or dyspnea	302/275/463/405/18.5/1012	ND
Sato et al. [18]	7/M/Japanese	+	ND	ND	ND	ND	ND	ND	ND/ND/ND/ND/ND/ND	
	2/F/Japanese	+	ND	ND	ND	ND	ND	ND	ND/ND/ND/ND/ND/ND	ND^b^
	2/F/Japanese	+	ND	ND	ND	ND	ND	ND	ND/ND/ND/ND/ND/ND	
Kobayashi et al. [3]	4/F/Japanese	+	+	ND	−	3 months	ND	cough or dyspnea	ND/ND/ND/ND/ND/ND	Anti-MDA5 (ND)
	6/F/Japanese	+	+	ND	+	5 months	ND	cough or dyspnea	ND/ND/ND/ND/ND/ND	Anti-MDA5 (ND)
	4/F/Japanese	+	+	ND	−	6 months	ND	cough or dyspnea	ND/ND/ND/ND/ND/ND	Anti-MDA5 (ND)
Hou et al. [19]	10/F/Chinese	+	ND	ND	+	2 months	ND	−	173/ND/419/588/ND/ND	Anti-MDA5 (ND)
Yeung et al. [6]	16/F/Chinese	+	+	+	−	2 months	1 month	cough	ND/64/407/858/ND/ND	Anti-MDA5 (ND)
Present case	2/F/Japanese	+	+	−	+	3 months	0 day	cough, dyspnea	69/49/576/22/13.5/3420	Anti-MDA5 (> 7500)

Reference	Chest CT findings	Myositis on femoral MRI	Respiratory support			Treatment	Outcome	Duration from of RP-ILD onset to death
			O2	CMV	ECMO			
Tosun et al. [9]	CABB	ND	ND	ND	ND	PSL, MPT, IVCY	Death	7 weeks
Zulian et al. [10]	CABB, GGO, SCS	ND	+	+	+	PSL, MPT, CPM	Alive	−
Nonaka et al. [3, 11]	CABB, SCS	+	+	−	−	MPT, PSL, CyA, IVIG	Alive	−
Nagai et al. [12]	CABB, GGO, TB, SCS	+	ND	ND	ND	PSL, MPT, CyA, IVCY, IVIG	Death	4 weeks
Sakurai et al. [13]	Pleural effusion	+	+	+	−	mPSL, MPT, CyA, IVCY, PE	Death	3 weeks
Kobayashi et al. [3, 8, 13, 15, 16]	GGO, SCS	ND	−	−	−	PSL, MPT, CyA, IVIG	Alive	−
	GGO	ND	+	ND	ND	PSL, MPT, AZP, CyA	Death	1 month
	GGO, SCS	ND	ND	ND	ND	PSL, MPT, CyA	Alive	−
Ishikawa et al. [3, 17]	CABB, GGO, SCS	+	+	+	−	PSL, MPT, CyA, IVIG	Death	7 weeks
Sato et al. [18]	GGO	ND	ND	ND	ND	PSL, MPT, CyA, IVIG	Death	< 4 months
	GGO	ND	ND	ND	ND	PSL, MPT, CyA, IVCY, IVIG	Death	< 4 months
	GGO	ND	ND	ND	ND	PSL, MPT, CyA, IVCY, IVIG	Death	< 4 months
Kobayashi et al. [3]	SCS	ND	ND	ND	ND	ND	Death	2 months
	SCS	ND	ND	ND	ND	ND	Death	2 months
	CABB, GGO, TB, SCS	ND	ND	ND	ND	ND	Death	1 month
Hou et al. [19]	CABB, GGO, SCS	ND	ND	ND	ND	mPSL, IVCY, IVIG	Death	2 months
Yeung et al. [6]	CABB, SCS	+	+	−	−	PSL, MPT, Tac, MMF, MTX, IVIG, IVCY, RTX	Alive	−
Present case	CABB, GGO, TB, SCS	+	+	+	+	PSL, MPT, CyA, Tac, MMF, IVCY, IVIG, PE, RTX	Alive	−

*M* Male, *F* Female, *JDM* Juvenile dermatomyositis, *ILD* Interstitial lung disease, *RP-ILD* Rapidly progressive interstitial lung disease, *CT* Computed tomography, *CABB* Consolidation around bronchovascular bundles, *SCS* Subpleural curvilinear shadow, *GGO* Ground-glass opacity, *TB* Traction bronchiectasis, *MRI* Magnetic resonance imaging, *O2* Oxygen, *CMV* Continuous mandatory ventilation, *ECMO* Extracorporeal membrane oxygenation, *PSL* Prednisolone, *MPT* Methylprednisolone pulse therapy, *IVCY* Intravenous cyclophosphamide, *CPM* Cyclophosphamide, *CyA* Cyclosporine A, *IVIG* Intravenous immunoglobulin, *MDA5* melanoma differentiation-association gene 5, *PE* Plasma exchange, *AZP* Azathioprine, *Tac* tacrolimus, *MMF* Mycophenolate mofetil, *MTX* Methotrexate, *RTX* Rituximab, *ND* No data

^a^The levels of anti-MDA5 antibody in each patient (range, 357–902 U). ^b^Two patients were positive for anti-MDA5 antibody (range, 78–172 U). One case was not tested for it

“dermatomyositis” [MeSH Terms] OR “dermatomyositis” [All Fields] OR (“juvenile” [All Fields] AND “dermatomyositis” [All Fields]) OR “juvenile dermatomyositis” [All Fields]) AND (“lung diseases, interstitial” [MeSH Terms] OR (“lung” [All Fields] AND “diseases” [All Fields] AND “interstitial” [All Fields]) OR “interstitial lung diseases” [All Fields] OR (“interstitial” [All Fields] AND “lung” [All Fields] AND “disease” [All Fields]) OR “interstitial lung disease” [All Fields]) AND (“infant” [MeSH Terms] OR “infant” [All Fields] OR “infants” [All Fields] OR “infant s” [All Fields] OR (“child” [MeSH Terms] OR “child” [All Fields] OR “children” [All Fields] OR “child s” [All Fields] OR “children s” [All Fields] OR “childrens” [All Fields] OR “childs” [All Fields]) OR (“paediatrics” [All Fields] OR “pediatrics” [MeSH Terms] OR “pediatrics” [All Fields] OR “paediatric” [All Fields] OR “pediatric” [All Fields]) OR (“adolescences” [All Fields] OR “adolescency” [All Fields] OR “adolescent” [MeSH Terms] OR “adolescent” [All Fields] OR “adolescence” [All Fields] OR “adolescents” [All Fields] OR “adolescent s” [All Fields]) OR (“juvenile” [All Fields] OR “juvenile s” [All Fields] OR “juveniles” [All Fields] OR “juvenility” [All Fields]).

Among the 18 patients (6 men and 12 women), the median age at disease onset was 6.5 (range, 2–16) years. There were 16 Asians, including 14 Japanese patients. Although 11 patients demonstrated apparent muscle weakness, 4 were classified as having juvenile clinically amyopathic dermatomyositis, whereas the other 3 had no available data. The median months of interval between the onset of JDM and diagnosis of ILD was 3 (range, 1–8) months. The median months of interval between the diagnoses of JDM and ILD was 1 month (range, 0–7 months). Upon the diagnosis of ILD, respiratory symptoms were observed in 11 patients, whereas 3 were asymptomatic. Furthermore, 12 patients were positive for anti-MDA5 antibody, 1 was positive for anti-Jo-1 antibody, and 5 patients were not tested for these. Two patients, including the patient whose case has been reported here, required ECMO. A total of 12 patients died despite recieving potent combination therapy with MPT, CyA, and/or, IVCY. The duration from ILD onset to death was within 4 months for all patients. ILD was relatively more severe in the present case than in previous cases. Among the six survivors, ventilation was not required in three and not noted in one. Ours is a rare case of a successful treatment of refractory RP-ILD that required VV-ECMO. There have been few reports on RTX use in severe cases of RP-ILD refractory to combination therapy.

Yeung et al. recently reported a case of RP-ILD with JDM treated with multiple agents, including RTX [6]. However, their patient exhibited much milder respiratory symptoms than our patient. Furthermore, because multiple agents were combined with RTX, it is difficult to evaluate the efficacy of RTX alone. However, the efficacy of RTX has been reported in many adult patients with dermatomyositis [5]. Although the mortality rate in dermatomyositis-associated RP-ILD has been reported to be approximately 50% within 6 months [20], its prognosis can be improved with early diagnosis and aggressive treatment, including combination therapy with/without RTX [5, 21]. In the present case, combination therapy, IVIG, and PE were inadequate to manage RP-ILD, but the patient gradually improved after the addition of RTX. This combination therapy also stabilized the anti-MDA-5 antibody level, which has previously been associated with RP-ILD/dermatomyositis [21, 22]. As mentioned earlier, it is challenging to evaluate the efficacy of RTX alone. We consider that clinical improvement in our patient was resulted from the combined effects of multiple immunosuppressive therapies, including IVCY and RTX. The possible addition of RTX in children with RP-ILD refractory to the aforementioned treatments must be considered promptly. However, RTX is not reimbursed in Japan for patients with JDM or ILD. Concerns regarding the use of RTX have been discussed within the medical team considering multiple reports on its adverse events in interstitial pneumonia [23]. In our case, we obtained informed consent from the parents of the patient and approval from the hospital’s ethics committee.

There have been a few reports on patients with dermatomyositis/JDM-associated RP-ILD who were treated with PE but had poor outcomes [4, 12, 24]. Some experts suggest that PE can be considered in patients who are unresponsive to combination therapy with corticosteroids and immunosuppressive agents [4]. In our case, the serum level of anti-MDA5 antibody decreased after frequent PE; however, it increased soon, and the patient’s respiratory failure worsened. Both RTX and PE have the same effect in terms of reducing autoantibody levels, but RTX suppresses antibody production more radically and persistently. In addition, RTX has the advantage of suppressing T and B cell interactions and increase of the number of regulatory T cells [25].

VV-ECMO can be indicated in cases of life-threatening respiratory failure, but the prognosis for adult patients with RP-ILD who require VV-ECMO is poor [4]. VV-ECMO is also used as a bridging therapy in lung transplantation for adult patients [5]. Our patient was successfully weaned off VV-ECMO 24 days after RTX administration. Zulian et al. have reported a similar case of a pediatric patient who survived after VV-ECMO [10]. Therefore, VV-ECMO should be considered as a supportive therapy in combination with immunosuppressive therapy, including RTX, in children with severe JDM and RP-ILD.

We took several precautions in the management of this critical patient. Trimethoprim-sulfadiazine was administered as prophylaxis for infection. We closely monitored our patient for the growth of *Pneumocystis carinii*, cytomegalovirus, and fungi. Deep sedation was also performed for stable respiratory management. We refrained from using high ventilatory pressures and tolerated hypoxemia and hypercapnia to some extent because high respiratory pressures may cause pneumothorax and pneumoperitoneum, which can result in sudden respiratory and circulatory collapse [26]. Placing the patient in the prone position might have also contributed to the improvement of respiratory status.

In conclusion, we treated a pediatric patient with life-threatening RP-ILD associated with JDM who ultimately required VV-ECMO. Initially, she showed resistance to multiple therapeutic strategies such as corticosteroids, immunosuppressive agents, and PE. Finally, the addition of RTX improved respiratory failure and managed anti-MDA-5 antibody level. Although further studies are warranted to validate our results, RTX may be a promising drug against RP-ILD associated with JDM. The efficacy of RTX in the early phase of ILD should also be studied in the future.

## Data Availability

The datasets used and/or analyzed during the current study are available from the corresponding author upon reasonable request.

## Abbreviations

CyA: Cyclosporine A
ILD: Interstitial lung disease
IVCY: Intravenous cyclophosphamide
IVIG: Intravenous immunoglobulin
JDM: Juvenile dermatomyositis
KL-6: Krebs von den Lungen-6 glycoprotein
MDA-5: Melanoma differentiation-association gene 5
MMF: Mycophenolate mofetil
MPT: Methylprednisolone pulse therapy
PE: Plasma exchange
PSL: Prednisolone
RP-ILD: Rapidly interstitial lung disease
RTX: Rituximab
VV-ECMO: Venovenous extracorporeal membrane oxygenation

## References

[1] Kobayashi I, Akioka S, Kobayashi N, Iwata N, Takezaki S, Nakaseko H (2012). Clinical practice guidance for juvenile dermatomyositis (JDM) 2018-Update. Mod Rheumatol.

[2] Kishi T, Miyamae T, Hara R, Nakajima S, Imagawa T, Mori M (2013). Clinical analysis of 50 children with juvenile dermatomyositis. Mod Rheumatol.

[3] Kobayashi N, Takezaki S, Kobayashi I, Iwata N, Mori M, Nagai K (2015). Clinical and laboratory features of fatal rapidly progressive interstitial lung disease associated with juvenile dermatomyositis. Rheumatology.

[4] Romero-Bueno F, Diaz Del Campo P, Trallero-Araguás E, Ruiz-Rodríguez JC, Castellvi I, Rodriguez-Nieto MJ (2020). Recommendations for the treatment of anti-melanoma differentiation- associated gene 5-positive dermatomyositis-associated rapidly progressive interstitial lung disease. Semin Arthritis Rheum.

[5] Huang K, Vinik O, Shojania K, Yeung J, Shupak R, Nimmo M (2019). Clinical spectrum and therapeutics in Canadian patients with anti-melanoma differentiation-associated gene 5 (MDA5)-positive dermatomyositis: a case-based review. Rheumatol int.

[6] Yeung TW, Cheong KN, Lau YL, Tse KN (2021). Adolescent-onset anti-MDA5 antibody-positive juvenile dermatomyositis rapidly progressive interstitial lung disease and spontaneous pneumomediastinum: a case report and literature review. Pediatr Rheumatol Online J.

[7] Kobayashi I, Okura Y, Yamada M, Kawamura N, Kuwana M, Ariga T (2011). Anti-melanoma differentiation-associated gene 5 antibody is a diagnostic and predictive marker for interstitial lung disease associated with juvenile dermatomyositis. J Pediatr.

[8] Ueki M, Kobayashi I, Takezaki S, Tozawa Y, Okura Y, Yamada M (2019). Myositis-specific autoantibodies in Japanese patients with juvenile idiopathic inflammatory myopathies. Mod Rheumatol.

[9] Tosun A, Serdaroğlu G, Aslan MT, Polat M, Akalin T, Tekgul H (2006). Severe juvenile dermatomyositis: two patients complicated with extra musculocutaneous involvement. Rheumatol int.

[10] Zulian F, Martinez Toledo MM, Amigoni A, Martini G, Agosto C (2007). Successful use of extracorporeal membrane oxygenation for severe interstitial lung disease in a child with dermatomyositis. Intensive Care Med.

[11] Nonaka Y, Imanaka K, Nerome Y, Maeno N, Akaike N, Arimura H (2008). A case of anti-Jo-1 antibody-positive juvenile dermatomyositis complicated by interstitial lung disease: successful treatment with cyclosporine A [in Japanese]. J jpn Pediatr Soc.

[12] Nagai Y, Mizuno T, Yoshizawa C, Ishikawa O (2010). Fatal interstitial pneumonia in juvenile dermatomyositis. Eur J Dermatol.

[13] Sakurai N, Nagai K, Tsutsumi H, Ichimiya S (2011). Anti-CADM-140 antibody-positive juvenile dermatomyositis with rapidly progressive interstitial lung disease and cardiac involvement. J Rheumatol.

[14] Kobayashi I, Okura Y, Yamada M, Kawamura N, Kuwana M, Ariga T (2011). Anti-melanoma differentiation-associated gene 5 antibody is a diagnostic predictive marker for interstitial lung disease associated with juvenile dermatomyositis. J Pediatr.

[15] Kobayashi I, Ono S, Kawamura N, Okano M, Miyazawa K, Shibuya H (2001). KL-6 is a potential marker for intestitial lung disease associated with juvenile dermatomyositis. J Pediatr.

[16] Kobayashi I, Yamada M, Takahashi Y, Kawamura N, Okano M, Sakiyama Y (2003). Interstitial lung disease associated with juvenile dermatomyosistis: clinical features and efficacy of cyclosporin A. Rheumatology (Oxford).

[17] Ishikawa J, Shikama Y, Takahashi E, Akagi K (2011). Rapidly progressive interstitial pneumoniae with juvenile dermatomyositis [in Japanese]. J Jpn Pediatr Soc.

[18] Sato S, Uejima Y, Nanbu M, Suganuma E, Takano T, Tanaka R (2017). Clinical analysis and outcome of interstitial lung disease complicated with juvenile dermatomyositis and juvenile polymyositis. Mod Rheumatol.

[19] Hou J, Zhou ZX, Li JG, Xu YJ, Ding YC (2019). Three cases report of juvenile dermatomyositis with positive anti-melanoma differentiation associated gene 5 (MDA5) antibody and severe interstitial lung disease and literature review. Zhonghua Er Ke Za Zhi.

[20] Ye S, Chen XX, Lu XY, Wu MF, Deng Y, Huang WQ (2007). Adult clinically amyopathic dermatomyositis with rapid progressive interstitial lung disease: a retrospective cohort study. Clin Rheumatol.

[21] Matsushita T, Mizumaki K, Kano M, Yagi N, Tennichi M, Takeuchi A (2017). Antimelanoma differentiation-associated protein 5 antibody level is a novel tool for monitoring disease activity in rapidly progressive interstitial lung disease with dermatomyositis. Br J Dermatol.

[22] Tsuji H, Nakashima R, Hosono Y, Imura Y, Yagita M, Yoshifuji H (2020). Multicenter Prospective Study of the Efficacy and Safety of Combined Immunosuppressive Therapy With High-Dose Glucocorticoid, Tacrolimus, and Cyclophosphamide in Interstitial Lung Diseases Accompanied by antimelanoma Differentiation-Associated Gene 5-Positive Dermatomyositis. Arthritis Rheumatol.

[23] Wagner SA, Mehta AC, Laber DA (2007). Rituximab-induced interstitial lung disease. Am J Hematol.

[24] Kagawa H, Tsujino K, Yamamoto Y, Iwai A, Hara R, Matsuki T (2020). Acute lung injury after plasma exchange in a patient with anti-MDA5 antibody-positive, rapidly progressive, interstitial lung disease: A case report. Respir Med Case Rep.

[25] Cooper N, Arnold DM (2010). The effect of rituximab on humoral and cell mediated immunity and infection in the treatment of autoimmune diseases. Br J Haematol.

[26] Anzueto A, Frutos-Vivar F, Esteban A, Alía I, Brochard L, Stewart T (2004). Incidence, risk factors and outcome of barotrauma in mechanically ventilated patients. Intensive Care Med.

