# Correction: Successful rituximab treatment for severe rapidly progressive interstitial lung disease with anti-MDA5 antibody-positive juvenile dermatomyositis: a case report and literature review

**DOI:** 10.1186/s12969-022-00736-0

**Published:** 2022-09-05

**Authors:** Kentaro Nishi, Masao Ogura, Naotaka Tamai, Masafumi Gima, Kentaro Ide, Goro Koinuma, Koichi Kamei, Shuichi Ito

**Affiliations:** 1 Division of Nephrology and Rheumatology, National Center for Child Health and Development, Tokyo, Japan; 2 Division of Pulmonology, National Center for Child Health and Development, Tokyo, Japan; 3 Division of Critical Care Medicine, National Center for Child Health and Development, Tokyo, Japan; 4 Department of Pediatrics, Yokohama City University Graduate School of Medicine, 3-9, Fukuura, Kanazawa-ku, Kanagawa, Yokohama 236-0004 Japan

**Correction: Pediatr Rheumatol 20, 60 (2022)**

**https://doi.org/10.1186/s12969-022-00723-5**

The original publication of this article [1] contained an incorrect author name. The incorrect and correct name are shown below. The original article has been updated.

**Author name**

- The incorrect author name is: Naofumi Gima
- The correct author name is: Masafumi Gima
